# Sleep Duration and Physical Activity as Predictors of Executive Function in Adolescents: A Longitudinal Study

**DOI:** 10.3390/brainsci16030302

**Published:** 2026-03-10

**Authors:** Rosa Ayuso-Moreno, Ana Rubio-Morales, Rubén Llanos-Muñoz, Tomás García-Calvo, Inmaculada González-Ponce

**Affiliations:** 1 Faculty of Sport Sciences, University of Extremadura, 10003 Cáceres, Spain; rmayusom@unex.es (R.A.-M.); rubiomorales@unex.es (A.R.-M.); 2 Faculty of Teacher Training, University of Extremadura, 10003 Cáceres, Spain; rubenlm@unex.es (R.L.-M.); ingopo@unex.es (I.G.-P.)

**Keywords:** executive functions, physical activity, sleep duration, inhibitory control, teenagers, longitudinal study, Fitbit, secondary school

## Abstract

**Highlights:**

**What are the main findings?**

- Objective physical activity predicted faster reaction times in inhibitory control and fewer lapses in sustained attention, whereas sleep duration showed no significant effects.
- In the low-activity subgroup, higher daily steps were unexpectedly associated with slower inhibitory control, possibly reflecting confounding factors or differential physiological adaptation.

**What are the implications of the main findings?**

- Habitual physical activity should be prioritised in educational settings to enhance adolescent cognitive efficiency.
- Future research must assess circadian timing and sleep variability, rather than relying solely on total sleep duration.

**Abstract:**

**Background/Objectives:** Adolescence is a critical period for executive function (EF) maturation. While sleep and physical activity (PA) are key lifestyle factors, their longitudinal impact on EF in ecologically valid settings is insufficiently characterised. This study examined the associations between objectively measured sleep duration, daily steps, and EF performance across one academic year (~9 months). **Methods:** A longitudinal study was conducted with 168 Spanish adolescents (13–16 years). Sleep duration and daily steps were monitored using Fitbit Charge 6 wearables for 7-day periods at baseline (M1; September 2024) and follow-up (M2; June 2025). EFs were assessed using three validated tasks: Stroop (inhibitory control), Psychomotor Vigilance Task-Brief (PVT-B; sustained attention), and Paced Auditory Serial Addition Test (PASAT; working memory). Linear Mixed Models (LMM) were employed to analyse the effects of the fixed factors (i.e., Group and Time), and their interactions. **Results:** PA, but not sleep duration, significantly predicted executive performance. The High_PA group demonstrated faster reaction times in inhibitory control (*p* = 0.007) and significantly fewer attentional lapses in sustained attention (*p* = 0.014). In contrast, sleep duration showed no significant main effects on EF domains (*p* > 0.05). Regression analyses confirmed that higher daily steps predicted faster reaction times in inhibitory control in the total sample (*r* = −0.173, *p* = 0.002), although an unexpected positive association was observed in the Low_PA group for inhibitory control, warranting cautious interpretation. **Conclusions:** These findings suggest that habitual PA is associated with better EF performance in adolescents, whereas sleep duration alone (without considering timing or variability) showed no significant associations with cognitive outcomes. Sensitivity analyses using clinically informed thresholds and continuous standardised predictors confirmed the robustness of these findings.

## 1. Introduction

Adolescence is a critical period for executive function (EF) maturation, during which the prefrontal cortex undergoes substantial refinement through synaptic pruning and myelination [1]. Sleep and physical activity have emerged as modifiable lifestyle factors that may shape neurocognitive trajectories, yet their longitudinal associations with EF remain insufficiently characterised in ecologically valid settings [2,3].

### 1.1. Sleep and Cognitive Function

Sleep is a fundamental pillar of cognitive homeostasis, supporting memory consolidation, synaptic plasticity, and metabolic restoration [4]. However, adolescents experience a biologically driven phase delay in circadian rhythms, resulting in a natural preference for later sleep and wake times [5,6]. Internationally, early school start times (typically 08:00–08:30) exacerbate this mismatch, with over 60% of adolescents failing to meet the recommended 8–10 h of sleep [7,8,9], potentially compromising cognitive readiness during morning hours.

Evidence suggests domain-specific vulnerability in sleep–cognition relationships. Meta-analytic findings indicate that sleep deficits significantly impair cognitive flexibility, while effects on inhibitory control and working memory remain inconsistent [10]. Acute sleep restriction primarily affects vigilance-related processing speed rather than selective attention [11].

A critical gap persists in the reliance on self-reported sleep measures, which show poor concordance with objective metrics [12] and in adolescent populations too [13]. Although consumer-grade wearables now enable continuous, objective monitoring in free-living conditions [14,15], few longitudinal studies have leveraged this technology to examine sleep–cognition associations across a full academic year.

### 1.2. Physical Activity and Executive Function

Beyond sleep, physical activity (PA) has emerged as another modifiable lifestyle factor consistently linked to enhanced cognitive performance in youth. International guidelines recommend that children and adolescents engage in an average of 60 min of moderate-to-vigorous physical activity daily [16], yet adherence rates remain suboptimal. This is concerning given that mental fatigue accumulation during sedentary school hours impairs both physical and cognitive performance [17,18].

Chronic PA—characterised by regular engagement over weeks or months (typically ≥4–6 weeks in intervention studies)—is hypothesised to confer stable neurobiological adaptations, including improved white matter integrity [19], increased cerebral blood flow, and upregulation of neurotrophic factors such as brain-derived neurotrophic factor (BDNF), a process driven by hemodynamic shear stress and metabolic demands during exertion [20,21]. These structural and functional changes are thought to enhance processing speed and executive control, particularly in tasks requiring sustained attention and inhibitory processes [22,23].

### 1.3. Current Study

Translation to real-world school settings remains challenging, as factors such as variability in engagement and competing curricular demands may attenuate laboratory-observed effects [24]. Moreover, PA and sleep are not independent: emerging evidence suggests that PA may function as a non-photic zeitgeber, strengthening circadian amplitude and improving sleep consolidation [25], thereby indirectly supporting cognitive performance through circadian entrainment. This underscores the need for longitudinal research in ecologically valid environments employing objective behavioural monitoring.

The present study therefore aimed to examine the longitudinal associations between objectively measured sleep duration, physical activity, and executive function performance in Spanish adolescents across one academic year under ecologically valid conditions. By doing so, this study sought to provide actionable evidence for educators and policymakers seeking to optimise adolescent learning environments. Crucially, we employed wearable technology (Fitbit Charge 6) to capture continuous behavioural data in free-living conditions, reflecting adolescents’ natural daily routines rather than controlled laboratory settings. We assessed three core executive function domains [26]: inhibitory control, sustained attention, and working memory. Given the domain-specific effects reported in prior meta-analyses [10] and the mechanistic plausibility of PA as a circadian modulator [25], we hypothesised that:

1. Higher sleep duration would predict better performance on tasks requiring cognitive flexibility and vigilance, with weaker or null associations for inhibitory control.
2. The sleep–EF relationship would be domain-specific, with stronger associations for vigilance-related outcomes than for inhibitory control or working memory.
3. Higher physical activity levels would predict faster reaction times, greater accuracy and fewer attentional lapses across all executive function domains.
4. The PA-EF relationship would be particularly pronounced in the high-activity subgroup, reflecting threshold effects consistent with neurobiological adaptation models.

## 2. Materials and Methods

### 2.1. Participants

A total of 168 Spanish adolescents (85 boys [weight: 65.84 ± 19.27 kg; height: 172.56 ± 8.24 cm] and 83 girls [weight: 57.16 ± 9.90 kg; height: 162.29 ± 6.60 cm]; *M*_age_ = 14.54 ± 0.73 years) from seven secondary schools in the region of Extremadura voluntarily participated in this longitudinal study. A priori power analysis (G*Power 3.1; [27]) indicated a minimum of 92 participants (f^2^ = 0.15, α = 0.05, power = 0.80); our sample (118–160 valid cases) exceeded this threshold. The inclusion criteria were: (a) enrolment in compulsory secondary education (ESO), (b) age between 13 and 16 years, and (c) willingness to wear a Fitbit device for 7-day monitoring periods at each assessment point (M1 and M2). Exclusion criteria included: (a) diagnosed sleep disorders, (b) neurological or psychiatric conditions affecting cognitive function, (c) use of medications known to affect sleep or cognition, and (d) inability to complete the cognitive assessment battery.

Complete cognitive data were available for 152–160 participants at baseline (M1) and 141–152 at follow-up (M2), depending on the measure. Fitbit-derived sleep data were available for 155 participants at M1 and 118 at M2, while physical activity data (daily steps) were available for 160 participants at M1 and 141 at M2.

The participants were informed about the study procedures, and written informed consent was obtained from parents or legal guardians, with verbal assent obtained from all participants. The study was conducted in accordance with the ethical principles outlined in the Declaration of Helsinki and was approved by the Bioethics Committee of the University of Extremadura (approval protocol number: 94//2024).

### 2.2. Outcomes and Instruments

#### 2.2.1. Sleep and Physical Activity Monitoring

Sleep duration was objectively assessed using Fitbit Charge 6 devices (Fitbit Inc., San Francisco, CA, USA) worn on the non-dominant wrist for 7 consecutive days at each measurement point. Sleep duration (hours per night) was automatically detected by the device’s multi-sensor algorithm combining photoplethysmography (PPG) and accelerometery. Although direct validation for the specific Charge 6 model is limited, validation studies of previous generations of this device series have demonstrated sensitivity exceeding 95% for detecting sleep periods in adolescent populations [14,28].

For analytical purposes, participants were classified into sleep groups based on the median split of average sleep duration: High Sleep (≥7.26 h) and Low Sleep (<7.26 h).

PA was objectively assessed using the same Fitbit Charge 6 devices worn for 7 consecutive days at each measurement point. Daily step count was used as the primary measure of physical activity. For analysis purposes, participants were classified into physical activity groups based on the median split of average daily steps: High Physical Activity (High_PA; ≥9650 steps/day) and Low Physical Activity (Low_PA; <9650 steps/day). The median split was chosen in the absence of validated population-specific thresholds, ensuring balanced group sizes for subsequent mixed model analyses. Importantly, group assignment was based on the overall median across both time points, ensuring that each participant remained in the same group at M1 and M2, thereby avoiding the classification instability associated with time-point-specific median splits.

#### 2.2.2. Executive Function Assessment

The Stroop Colour-Word Task was used to assess inhibitory control [29,30]. Participants were presented with colour words (e.g., “RED”) displayed in incongruent ink colours (e.g., blue) and were required to name the ink colour while ignoring the word meaning. This task requires the inhibition of the automatic response to read the word itself, thereby taxing executive control processes. Reaction time (ms) and accuracy (%) were recorded as outcome measures over a 1 min administration.

The Brief Psychomotor Vigilance Task (PVT-B) was employed to assess sustained attention and vigilance [31]. This 3 min task required participants to respond as quickly as possible when a visual stimulus appeared at random intervals (1–4 s). Mean reaction time (ms) and number of lapses (responses >500 ms) were recorded as outcome measures.

The Paced Auditory Serial Addition Test (PASAT) was used to assess working memory and information processing speed [32]. Participants heard single-digit numbers presented every 3 s and were required to add each number to the one immediately preceding it. This 3 min task places demands on working memory, attention, and arithmetic processing. Reaction time (ms) and accuracy (%) were recorded as outcome measures.

### 2.3. Procedures

A longitudinal observational design was carried out over a complete academic year (September 2024 to June 2025). Data were collected at two time points: baseline (M1, September 2024) and follow-up (M2, June 2025). At each time point, participants wore Fitbit Charge 6 devices for 7 consecutive days [33,34] and completed the cognitive assessment battery in a quiet classroom setting. Cognitive tests were administered using the SOMA NPT application (version 13.0; SOMA Analytics, Lucerne, Switzerland) on iPad devices in the same order across all participants and time points. For the duration of the study, participants were encouraged to maintain their usual daily habits.

### 2.4. Statistical Analysis

Data preprocessing and statistical analyses were performed using a modern data science framework in Python (version 3.12). The analytical pipeline utilised *pandas* library for data manipulation and the *statsmodels* and *scipy* packages for advanced longitudinal modelling, ensuring reproducibility and robustness in the handling of large dataset structures. Linear Mixed Models (LMM) were employed as the primary analytical procedure. LMMs are particularly robust for handling unbalanced longitudinal data and missing observations, providing a more flexible alternative to traditional repeated measures ANOVA [35].

The modelling strategy included both fixed and random effects. Specifically, Group (High vs. Low Physical Activity; or High vs. Low Sleep Duration) and Time (M1 vs. M2) were included as fixed factors, along with their interaction term (Group × Time). To account for the non-independence of repeated measures and the inherent inter-individual variability, Participant ID was included as a random intercept in all models. While lifestyle factors were also analysed as continuous predictors in supplementary regressions, the median-split grouping was maintained in the primary LMM to examine specific threshold effects and differential developmental trajectories between high- and low-adherence subgroups [36]. The median-split approach was retained to facilitate interpretability and to explore potential non-linear or threshold-like patterns in real-world behavioural adherence, while complementary continuous analyses were conducted to mitigate information loss. Prior to the main analysis, a variance component analysis confirmed that the Intraclass Correlation Coefficient (ICC) exceeded 10% for all dependent variables, corroborating the necessity of treating participants as a random effect [36]. Model comparison using AIC confirmed that models with fixed factors (Group, Time) and interactions provided optimal fit for most executive function outcomes.

Post hoc comparisons were conducted to obtain estimated marginal means (*EMMeans*) and to identify specific differences between groups at each time point. Results from LMM are presented as coefficients and standard error (Coef ± SE). Additionally, to further explore the continuous relationship between lifestyle habits and cognitive outcomes, linear regression analyses were performed. These results are reported as Pearson’s correlation coefficients (*r*) and unstandardised regression coefficients, where *β*_0_ represents the intercept and *β*_1_ indicates the slope of the regression line.

Statistical significance was set at *p* < 0.05. Finally, the magnitude of the observed differences was quantified using Cohen’s d effect sizes [37]. Following conventional criteria, effect sizes were interpreted as small (0.20–0.49), medium (0.50–0.79), and large (≥0.80).

To assess the robustness of findings to the choice of grouping criterion, supplementary sensitivity analyses were conducted using (a) clinically informed thresholds (≥10,000 steps/day for physical activity [38,39]; ≥8 h/night for sleep duration [9]) and (b) continuous standardised predictors (z-scores) entered directly into LMMs. Model comparison using AIC confirmed that models with categorical fixed factors provided optimal fit for most outcomes, supporting the median-split approach as the primary analysis.

## 3. Results

Table 1 shows the descriptive statistics and main effects analyses of the LMM for executive function outcomes by sleep duration group. Regarding lifestyle variables across the sample, sleep duration decreased from M1 (7.47 ± 0.83 h) to M2 (7.06 ± 0.82 h). The median split for sleep duration was 7.26 h/night.

Sleep duration groups did not show significant main effects on any executive function measure. The main effect of Group was non-significant for Stroop RT (*F*(1, 307) = 0.81, *p* = 0.369; *R*^2^_m_ = 0.241, *R*^2^_c_ = 0.589), PVT-B RT (*F*(1, 303) = 0.01, *p* = 0.916; *R*^2^_m_ = 0.286, *R*^2^_c_ = 0.689), PVT-B lapses (*F*(1, 300) = 1.13, *p* = 0.288; *R*^2^_m_ = 0.243, *R*^2^_c_ = 0.618), PASAT RT (*F*(1, 306) = 0.93, *p* = 0.336; *R*^2^_m_ = 0.431, *R*^2^_c_ = 0.804), and PASAT accuracy (*F*(1, 306) = 0.89, *p* = 0.344; *R*^2^_m_ = 0.362, *R*^2^_c_ = 0.743).

Significant main effects of Time were observed for Stroop RT (*F*(1, 307) = 10.66, *p* = 0.001, *R*^2^_m_ = 0.240, *R*^2^_c_ = 0.585), PASAT RT (*F*(1, 306) = 117.43, *p* < 0.001, *R*^2^_m_ = 0.433, *R*^2^_c_ = 0.807), and PASAT accuracy (*F*(1, 306) = 57.10, *p* < 0.001, *R*^2^_m_ = 0.358, *R*^2^_c_ = 0.745), primarily reflecting practice effects. No significant Group × Time interactions were found. Post-hoc comparisons for sleep duration groups are presented in Table 2.

**Table 2:** Multiple comparisons (intra- and inter-group) for executive function outcomes by sleep duration group.

Variable	Type	Comparison	*p*	*d*	CI (95%)
Stroop RT (ms)	Inter-group (M1)	High_Sleep vs. Low_Sleep	0.433	0.12	[−0.19, 0.43]
	Inter-group (M2)	High_Sleep vs. Low_Sleep	0.701	0.06	[−0.26, 0.38]
	Intra-group	M1 vs. M2 (High_Sleep)	<0.001 ***	−0.42	[−0.75, −0.09]
	Intra-group	M1 vs. M2 (Low_Sleep)	0.038 *	−0.28	[−0.60, 0.05]
Stroop Acc (%)	Inter-group (M1)	High_Sleep vs. Low_Sleep	0.325	−0.16	[−0.47, 0.15]
	Inter-group (M2)	High_Sleep vs. Low_Sleep	0.709	0.06	[−0.26, 0.38]
	Intra-group	M1 vs. M2 (High_Sleep)	0.868	−0.02	[−0.34, 0.30]
	Intra-group	M1 vs. M2 (Low_Sleep)	0.052	−0.18	[−0.51, 0.14]
PVT-B RT (ms)	Inter-group (M1)	High_Sleep vs. Low_Sleep	0.824	−0.04	[−0.35, 0.28]
	Inter-group (M2)	High_Sleep vs. Low_Sleep	0.938	0.01	[−0.31, 0.33]
	Intra-group	M1 vs. M2 (High_Sleep)	0.185	−0.14	[−0.47, 0.18]
	Intra-group	M1 vs. M2 (Low_Sleep)	0.401	−0.10	[−0.43, 0.23]
PVT-B Lapses (n)	Inter-group (M1)	High_Sleep vs. Low_Sleep	0.311	−0.16	[−0.48, 0.15]
	Inter-group (M2)	High_Sleep vs. Low_Sleep	0.818	0.04	[−0.28, 0.36]
	Intra-group	M1 vs. M2 (High_Sleep)	0.144	0.16	[−0.17, 0.49]
	Intra-group	M1 vs. M2 (Low_Sleep)	0.748	−0.04	[−0.38, 0.29]
PASAT RT (ms)	Inter-group (M1)	High_Sleep vs. Low_Sleep	0.464	−0.12	[−0.43, 0.19]
	Inter-group (M2)	High_Sleep vs. Low_Sleep	0.109	−0.26	[−0.58, 0.06]
	Intra-group	M1 vs. M2 (High_Sleep)	<0.001 ***	−1.05	[−1.42, −0.69]
	Intra-group	M1 vs. M2 (Low_Sleep)	<0.001 ***	−0.95	[−1.31, −0.59]
PASAT Acc (%)	Inter-group (M1)	High_Sleep vs. Low_Sleep	0.476	0.11	[−0.20, 0.42]
	Inter-group (M2)	High_Sleep vs. Low_Sleep	0.020 *	0.38	[0.06, 0.71]
	Intra-group	M1 vs. M2 (High_Sleep)	<0.001 ***	0.96	[0.60, 1.32]
	Intra-group	M1 vs. M2 (Low_Sleep)	<0.001 ***	0.59	[0.25, 0.93]

*Note. d* = Cohen’s d; CI = confidence interval. Bold values indicate statistically significant effects (*p* < 0.05). * *p* < 0.05, *** *p* < 0.001.

**Figure 1:**
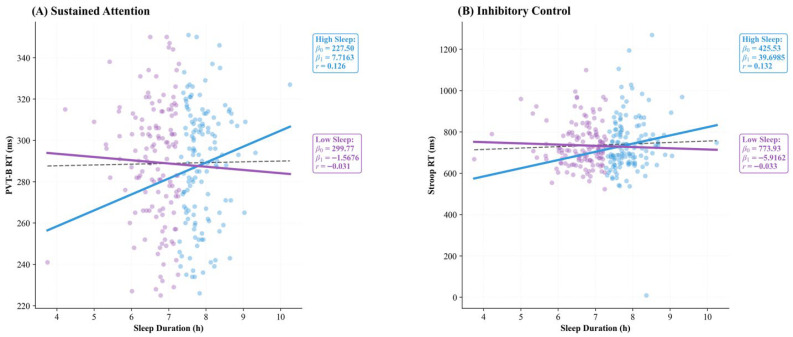
Linear regression analysis between sleep duration and executive function. (**A**) Sustained attention (PVT-B RT) and (**B**) inhibitory control (Stroop RT) showed no significant associations with sleep duration in the total sample or subgroups (all *p* > 0.05). Dashed line = total sample; solid lines = group-specific regressions. High_Sleep ≥ 7.26 h/night; Low_Sleep < 7.26 h/night. Regression statistics (*β*_0_, *β*_1_, *r*) are displayed within each panel. Note: *β*_0_ = intercept; *β*_1_ = slope.

Linear regression analyses further corroborated the findings from the LMM, demonstrating an absence of a significant predictive relationship between sleep duration and cognitive performance (*p* > 0.05). As illustrated in Figure 1, the regression lines for both the total sample and the stratified groups (High_Sleep vs. Low_Sleep) remained remarkably flat for both sustained attention (Figure 1A; Total *r* = 0.011) and inhibitory control (Figure 1B; Total *r* = 0.046), indicating that sleep duration was not a primary driver of executive performance in this sample.

Given the absence of significant sleep duration effects on executive function performance, we next examined whether physical activity showed differential associations with EF outcomes. Table 3 shows the descriptive statistics and main effects analyses of the LMM for executive function outcomes by physical activity group. Regarding lifestyle variables across the sample daily steps decreased (from 10,927 ± 3450 to 8880 ± 3863 steps/day). The median split for physical activity groups was 9650 steps/day.

A significant main effect of Group was found for Stroop RT (*F*(1, 307) = 7.40, *p* = 0.007), with the Low_PA group showing longer reaction times than the High_PA group. However, post hoc comparisons (Table 4) showed this effect was significant at M1 (*p* = 0.024) but not at M2 (*p* = 0.138), indicating the group difference was stronger at baseline. The model explained 25.3% of the variance through fixed effects (*R*^2^_m_ = 0.253) and 59.5% when including random effects (*R*^2^_c_ = 0.595). A significant main effect of Group was also found for PVT-B lapses (*F*(1, 300) = 6.05, *p* = 0.014), indicating that less active adolescents committed more attentional lapses. The model explained 24.7% of the variance through fixed effects (*R*^2^_m_ = 0.247) and 60.8% when including random effects (*R*^2^_c_ = 0.608).

For PASAT accuracy, a significant Group effect emerged (*F*(1, 306) = 4.08, *p* = 0.043), with the High_PA group showing higher accuracy. The model explained 36.4% of the variance through fixed effects (*R*^2^_m_ = 0.364) and 74.4% when including random effects (*R*^2^_c_ = 0.744). Significant main effects of Time were observed for PASAT RT (*F*(1, 306) = 114.41, *p* < 0.001; *R*^2^_m_ = 0.432, *R*^2^_c_ = 0.802) and PASAT accuracy (*F*(1, 306) = 42.38, *p* < 0.001), reflecting substantial practice effects. No significant Group × Time interactions were found, although substantial practice effects were observed for PASAT measures (*p* < 0.001).

**Figure 2:**
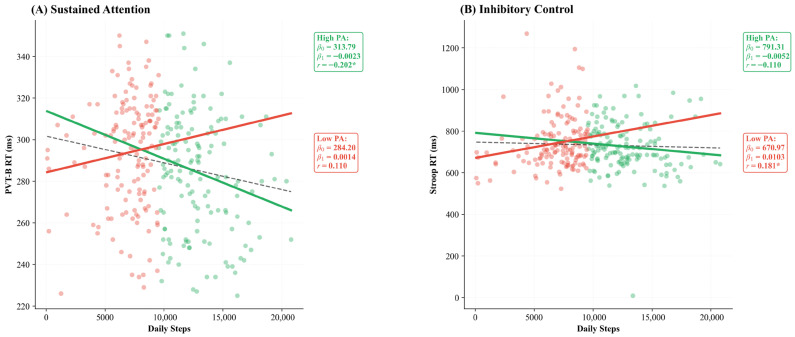
Linear regression analysis between daily steps and executive function. (**A**) Higher daily steps predicted faster sustained attention (PVT-B RT) in the total sample (*r* = −0.173, *p* = 0.002), driven by the High_PA group (*p* = 0.014). (**B**) For inhibitory control (Stroop RT), a significant positive association emerged only in the Low_PA group (*r* = 0.181, *p* = 0.027). Dashed line = total sample; solid lines = group-specific regressions. High_PA ≥ 9650 steps/day; Low_PA < 9650 steps/day. Regression statistics (*β*_0_, *β*_1_, *r*) are displayed within each panel. Note: *β*_0_ = intercept; *β*_1_ = slope; * *p* < 0.05.

Beyond these longitudinal trends, linear regression analyses revealed that higher daily steps significantly predicted faster reaction times in the total sample (*r* = −0.173 **, *p* = 0.002, *β*_1_ = −0.0016). However, this benefit was specifically driven by the High_PA group (*β*_1_= −0.0023, *p* = 0.014), as shown in Figure 2A. Conversely, for inhibitory control (Stroop RT), a significant positive correlation emerged only within the Low_PA group (*r* = 0.181, *p* = 0.027, *β*_1_ = 0.0103), suggesting that adolescents with lower activity levels exhibit an unexpected increase in reaction time with higher daily steps (Figure 2B).

Sensitivity analyses confirmed the robustness of the primary findings. With clinically informed thresholds (≥10,000 steps/day; ≥8 h/night), the PA group effect on Stroop RT remained significant (*p* = 0.042). With continuous standardised predictors (z-scores), daily steps significantly predicted Stroop RT (β = −19.41, *p* = 0.037), PVT-B RT (β = −6.11, *p* = 0.007), and PVT-B Lapses (β = −3.92, *p* < 0.001), while sleep duration showed no significant associations (all *p* > 0.68). These converging results confirm that the PA–EF associations are not attributable to the choice of grouping criterion.

## 4. Discussion

The present longitudinal study examined the associations between objectively measured sleep duration, PA and EF performance in Spanish adolescents across one academic year. Our findings revealed a clear divergence: physical activity, but not sleep duration, significantly predicted executive performance, particularly in domains of inhibitory control and sustained attention. These results provide nuanced insights into the modifiable lifestyle factors that may support cognitive development during the critical window of adolescence. Importantly, these findings were observed under ecologically valid school conditions, where behavioural variability, competing academic demands, and imperfect adherence are the norm rather than the exception.

### 4.1. Sleep Duration and Executive Function: Interpreting Null Findings

Sleep duration, averaged across 7-day periods, did not predict executive function performance. Taken together, these findings suggest that total sleep duration, when averaged across several nights, may represent an insufficient proxy to capture the sleep-related processes most relevant to executive functioning during adolescence. This null finding aligns with the domain-specific vulnerability framework [10] which indicates that cognitive shifting is more sensitive to sleep loss than inhibitory control or working memory. Our null effects for Stroop and PASAT are consistent with this framework. Critically, these findings should not be interpreted as evidence that sleep is unimportant for cognition, but rather that duration alone may be an insufficient metric.

Several methodological factors may explain these null findings regarding sleep duration. First, our focus on mean sleep duration may have obscured the effects of night-to-night variability and circadian misalignment (e.g., social jetlag). Research indicates that high variability in sleep patterns and misalignment between biological and social rhythms impair executive function and processing speed independently of total sleep time [40,41,42]. Furthermore, experimental evidence suggests that circadian misalignment specifically degrades sustained attention and cognitive throughput, effects that are not captured by aggregate duration metrics [43]. Second, the sample’s restricted range (M1: 7.47 ± 0.83 h) represents mild insufficiency; populations with severe restriction (<6 h) show stronger cognitive deficits [44]. Third, ceiling effects in accuracy measures (>98% for Stroop) may have masked subtle impairments. Finally, unmeasured factors (time-since-awakening, caffeine use, screen time) may have modulated sleep–cognition relationships beyond duration alone [45,46].

### 4.2. Physical Activity as a Robust Predictor of Executive Performance

In stark contrast to sleep, physical activity emerged as a significant predictor of multiple executive function outcomes. The High_PA group demonstrated faster reaction times in inhibitory control (Stroop RT: *p* = 0.007) and fewer attentional lapses (PVT-B: *p* = 0.014), alongside higher working memory accuracy (PASAT: *p* = 0.043). These findings align with theoretical frameworks, such as the exercise-induced neuroplasticity model [47], proposing that chronic PA facilitates neurobiological adaptations and enhances neural efficiency [48]. Recent evidence published in this journal further corroborates that physical activity induces structural brain adaptations essential for executive development [49].

A central finding was the association between daily steps and reaction time, which showed a linear pattern in the total sample. In the total sample, higher daily activity significantly predicted faster reaction times in sustained attention tasks (*r* = −0.173, *p* = 0.002), a relationship primarily driven by the High_PA group (β_1_ = −0.0023, *p* = 0.014; Figure 2A). This pattern suggests that adolescents who habitually engage in higher levels of physical activity develop more efficient neural processing [50,51]. Furthermore, regular engagement in physical activity improves pacing strategies and effort regulation in youth, specifically through the maturation of metacognitive monitoring and planning [52]. Reaction time is a sensitive marker of neurodevelopmental integrity, indexing the efficiency of white matter maturation and myelination during the adolescent period [53,54]. The observed association corroborates meta-analytic evidence that PA interventions preferentially enhance processing speed and executive control in youth [55].

It is important to contextualise the practical significance of these associations. The correlation between daily steps and PVT-B reaction time (*r* = −0.173) corresponds to approximately 3% of shared variance, and Cohen’s d values for PA group comparisons ranged from small to moderate (*d* = 0.24–0.37). These modest effect sizes are consistent with the broader literature on lifestyle–cognition associations in youth, where small but consistent effects carry cumulative public health significance [23].

An important consideration is the potential for reverse causation: adolescents with better executive function may be more successful in maintaining regular physical activity through superior self-regulation and planning. This bidirectional relationship has been documented in adults [56] and is increasingly recognized in youth, where executive control facilitates the adherence to complex activity behaviours [57]. Our observational design cannot disentangle whether PA improves EF, EF facilitates PA adherence, or both processes operate reciprocally. Additionally, third-variable confounding (e.g., family support, socioeconomic resources) may explain observed associations independent of any causal pathway [58,59].

An unexpected finding was a positive correlation between daily steps and Stroop RT in the Low_PA group (*r* = 0.181, *p* = 0.027; Figure 2B), indicating that within the less active subgroup, higher step counts were associated with slower inhibitory control. This small effect should be interpreted with considerable caution, as the Low_PA group encompasses substantial heterogeneity (ranging from ~4000 to ~9650 steps/day), and confounding variables not included in the model, such as body mass index, screen time, or socioeconomic status, may account for this pattern. Several non-exclusive explanations remain plausible: (1) unaccustomed exertion in poorly conditioned individuals may temporarily compromise cognitive resources [60], (2) insufficient neurobiological scaffolding to benefit from irregular activity bursts [24], or (3) competing demands when combining increased activity with insufficient recovery [61], or (4) unmeasured third variables that covary with both moderate step counts and slower reaction times within this subgroup. Notably, the sensitivity analysis using the 10,000-step clinical threshold yielded a comparable pattern (*r* = 0.155, *p* = 0.048), suggesting that this association is not an artefact of the specific median-split criterion. Nonetheless, given the modest effect size (*r*^2^ ≈ 3%) and observational design, we refrain from attributing this pattern to any single causal mechanism. This pattern aligns with U-shaped models where moderate, habitual PA confers benefits, while both inactivity and erratic exertion may be suboptimal [62].

The absence of significant Group × Time interactions suggests that the PA-EF relationship remained stable across the academic year, despite the observed decline in average daily steps from M1 to M2 (10,927 to 8880 steps/day). This secular trend likely reflects increased academic demands [63] and reduced outdoor activity during winter months [64]. Notably, even within this context of declining activity, between-group differences persisted, underscoring the resilience of habitual PA effects on cognitive performance. It should be noted that daily step counts primarily reflect behavioural adherence to physical activity recommendations and may not fully capture qualitative aspects such as intensity distribution or contextual characteristics of movement. Importantly, this pattern should not be interpreted as a detrimental effect of physical activity per se, but rather as a potential mismatch between acute cognitive demands and the physiological adaptation levels of habitually low-active adolescents.

### 4.3. Strengths, Limitations and Future Directions

This study advances the field by integrating consumer-grade wearable technology with longitudinal cognitive assessment in an ecologically valid school setting. The Fitbit Charge 6 provided objective monitoring of sleep and physical activity at two assessment points across one academic year, circumventing the recall bias inherent in self-report measures. Recent large-scale analyses confirm significant discrepancies between youth-reported and Fitbit-derived sleep data, underscoring the necessity of objective monitoring for accurate cognitive profiling [13]. Validation studies of earlier Fitbit models (Charge 2, Charge 4) have demonstrated sensitivity exceeding 95% for sleep detection and strong concordance with research-grade actigraphy for step counting [14,15]. While the Charge 6 employs refined photoplethysmography (PPG) and accelerometery algorithms, direct validation against polysomnography remains limited. Nevertheless, for population-level research prioritising ecological validity over laboratory precision, these devices represent a viable translational tool [28].

Our use of Linear Mixed Models (LMM) to handle longitudinal data with missing observations represents a methodological strength. LMMs account for the non-independence of repeated measures and inter-individual variability, providing more robust estimates than traditional repeated-measures ANOVA [35]. The high conditional *R*^2^ values (ranging from 0.589 to 0.804) indicate that participant-level random effects captured substantial variance, corroborating the necessity of this analytical approach. Future iterations could extend these models to include time-varying covariates (e.g., daily stress, academic load) and explore non-linear trajectories using growth curve modelling.

Despite these methodological strengths, several limitations warrant consideration. First, the median-split approach to creating activity and sleep groups, while common in exploratory research, imposes artificial thresholds that may not reflect true functional categories. However, three lines of evidence support the robustness of our findings: (a) model comparison using AIC confirmed that categorical grouping provided optimal fit for most outcomes, (b) sensitivity analyses with clinically informed thresholds (≥10,000 steps/day; ≥8 h/night) replicated the primary results, and (c) continuous standardised predictors (z-scores) confirmed significant PA–EF associations independently of any grouping criterion. Future studies should employ person-cantered methods (e.g., latent profile analysis) to identify naturally occurring subgroups based on multivariate behavioural patterns.

Second, our reliance on sleep duration as the sole sleep metric neglects critical dimensions such as sleep timing (bedtime/wake time consistency), sleep efficiency (percentage of time in bed asleep), and sleep architecture (REM/NREM proportions). Fitbit devices capture these data, and future analyses should leverage them to test domain-specific hypotheses (e.g., REM sleep predicting cognitive flexibility; deep sleep predicting working memory consolidation). Notably, the sensitivity analysis with the 8 h clinical threshold revealed a significant effect on PASAT RT (*p* = 0.003) that was absent with the median split, suggesting that clinically meaningful sleep categories may have greater predictive value than sample-derived thresholds for specific cognitive domains.

Third, the observational design precludes causal inference. While our longitudinal approach strengthens temporal precedence claims, experimental manipulation (e.g., randomised PA interventions) is necessary to establish causality. Future research should also examine whether PA-EF relationships differ fundamentally between habitually active and sedentary youth. Ongoing trials in Spanish schools, such as the MOVESCHOOL study [65], will complement our findings by testing intervention efficacy. Additionally, substantial practice effects, particularly for the PASAT (Cohen’s d up to 1.07), may have obscured subtle lifestyle-related plasticity. Although the 9-month interval is long, task familiarity likely contributed to performance gains; yet accuracy at M2 (86%) remained below ceiling, indicating learning had not plateaued. Future studies should incorporate familiarisation sessions to attenuate this effect.

Fourth, we did not control potential mediators (e.g., BDNF levels, cortisol reactivity, screen time) or moderators (e.g., pubertal status, socioeconomic status, body composition) that could refine our understanding of PA-EF mechanisms. Multi-modal designs integrating biomarkers, neuroimaging, and ecological momentary assessment would provide mechanistic insights beyond behavioural correlations. Furthermore, previous research indicates that emotional intelligence and academic burnout significantly mediate the relationship between school performance and well-being [66,67], variables that could interact with the lifestyle factors assessed here.

Finally, the generalisability of our findings may be limited to Spanish adolescents in regions with similar school schedules and cultural norms around sleep and PA. Cross-cultural replications are essential, particularly in contexts with delayed school start times or different PA infrastructure.

### 4.4. Practical Applications

The robust PA-EF associations documented here carry direct implications for secondary education policy in Spain and beyond. Given that Spanish adolescents spend up to 78% of their school day sedentary [65,68], integrating movement-based interventions into the academic schedule may yield cognitive dividends without sacrificing instructional time. Evidence-based strategies include:

1. **Active breaks**: Implementation of brief (5–10 min) bouts of moderate-intensity PA between lessons. Mechanistically, these bouts enhance cerebral blood flow and neural efficiency, facilitating the restoration of attentional resources depleted by prolonged cognitive effort [69]. Empirical evidence confirms that such breaks significantly improve students’ “on-task” behaviour and academic engagement [70,71], serving as an effective countermeasure against the mental load accumulation typically observed in school settings [17].
2. **Active commuting**: Promoting walking or cycling to school as a daily PA opportunity that also serves as a circadian zeitgeber [72].
3. **Curriculum-integrated movement**: Incorporating kinaesthetic learning activities (e.g., movement-based mnemonics, standing desks) into lesson plans. Recent meta-analyses indicate that integrating physical activity directly with academic content yields greater academic and behavioural benefits than non-integrated movement, likely through embodied cognition mechanisms [71,73].

While our data do not permit direct comparison of different PA intensities or frequencies, the consistent association between habitual daily step volume and cognitive performance, confirmed across median-split, clinical-threshold, and continuous-predictor analyses, aligns with emerging evidence that total daily movement volume, including light-to-moderate activity [74], may be a meaningful predictor of neurocognitive outcomes in youth [38]. Intervention programmes should therefore consider promoting consistent daily activity alongside intensity-focused targets, focusing on sustainable behaviour change rather than short-term performance gains [75,76].

## 5. Conclusions

This longitudinal study demonstrates that habitual physical activity is associated with better executive function performance in Spanish adolescents, particularly in inhibitory control and sustained attention. In contrast, sleep duration averaged across 7-day periods showed no significant associations with EF outcomes. These null findings likely reflect the complexity of sleep–cognition relationships, which extend beyond total duration to encompass sleep timing, night-to-night variability, and circadian alignment. Future research should expand beyond sleep duration to examine circadian alignment and sleep architecture, and randomised controlled trials are needed to establish whether the observed PA-EF associations reflect causal relationships that can be leveraged to enhance adolescent cognitive development. Taken together, these results suggest that under real-life school conditions, habitual physical activity emerges as a more robust and actionable correlate of executive functioning than sleep duration alone.

## Figures and Tables

**Table 1:** LMM descriptive statistics and main effects analyses for executive function outcomes by sleep duration group.

Variable	Group	M1 (Emmean ± SE)	M2 (Emmean ± SE)	Main Effect	*F*	*p*	*η* ^2^ *p*
Stroop RT (ms)	High_Sleep	760.67 ± 13.70	715.38 ± 11.90	Time	10.66	**0** .001 **	0.034
	Low_Sleep	743.58 ± 16.82	709.26 ± 10.54	Group	0.81	0.369	0.003
				Group × Time	0.35	0.555	0.001
Stroop Acc (%)	High_Sleep	98.72 ± 0.30	98.61 ± 0.37	Time	0.07	0.796	0.000
	Low_Sleep	99.22 ± 0.41	98.42 ± 0.33	Group	0.79	0.373	0.003
				Group × Time	1.53	0.217	0.005
PVT-B RT (ms)	High_Sleep	289.29 ± 3.32	286.92 ± 3.29	Time	1.18	0.278	0.004
	Low_Sleep	290.35 ± 3.35	286.58 ± 2.95	Group	0.01	0.916	0.000
				Group × Time	0.00	0.971	0.000
PVT-B Lapses (n)	High_Sleep	16.09 ± 1.59	18.28 ± 1.43	Time	1.91	0.167	0.006
	Low_Sleep	18.46 ± 1.71	17.82 ± 1.40	Group	1.13	0.288	0.004
				Group × Time	1.55	0.213	0.005
PASAT RT (ms)	High_Sleep	2056.04 ± 36.49	1745.71 ± 30.13	Time	117.43	<0.001 ***	0.277
	Low_Sleep	2093.46 ± 35.63	1817.37 ± 32.74	Group	0.93	0.336	0.003
				Group × Time	0.46	0.497	0.002
PASAT Acc (%)	High_Sleep	76.38 ± 1.57	88.92 ± 1.25	Time	57.10	<0.001 ***	0.157
	Low_Sleep	74.49 ± 2.11	83.69 ± 1.84	Group	0.89	0.344	0.003
				Group × Time	0.89	0.345	0.003

*Note.* High_Sleep = High Sleep Duration group; Low_Sleep = Low Sleep Duration group. Bold values indicate statistically significant effects (*p* < 0.05). ** *p* < 0.01, *** *p* < 0.001.

**Table 3:** LMM descriptive statistics and main effects analyses for executive function outcomes by physical activity group.

Variable	Group	M1 (Emmean ± SE)	M2 (Emmean ± SE)	Main Effect	*F*	*p*	*η* ^2^ *p*
Stroop RT (ms)	High_PA	727.81 ± 14.90	700.70 ± 10.17	Time	3.08	0.079	0.010
	Low_PA	776.85 ± 15.43	724.25 ± 12.12	Group	7.40	0.007 **	0.024
				Group × Time	2.69	0.101	0.009
Stroop Acc (%)	High_PA	98.52 ± 0.48	98.30 ± 0.37	Time	0.27	0.604	0.001
	Low_PA	99.44 ± 0.15	98.73 ± 0.32	Group	3.17	0.075	0.010
				Group × Time	0.80	0.370	0.003
PVT-B RT (ms)	High_PA	286.60 ± 3.19	282.88 ± 3.15	Time	1.83	0.176	0.006
	Low_PA	293.21 ± 3.44	290.72 ± 3.03	Group	2.05	0.152	0.007
				Group × Time	0.15	0.695	0.001
PVT-B Lapses (n)	High_PA	14.72 ± 1.39	16.27 ± 1.31	Time	1.12	0.291	0.004
	Low_PA	19.92 ± 1.85	19.87 ± 1.48	Group	6.05	0.014 *	0.020
				Group × Time	0.62	0.430	0.002
PASAT RT (ms)	High_PA	2047.41 ± 37.88	1738.04 ± 32.32	Time	114.41	<0.001 ***	0.272
	Low_PA	2103.25 ± 33.86	1826.32 ± 30.15	Group	1.89	0.169	0.006
				Group × Time	0.11	0.740	0.000
PASAT Acc (%)	High_PA	77.62 ± 1.66	88.00 ± 1.41	Time	42.38	<0.001 ***	0.122
	Low_PA	73.18 ± 2.04	84.58 ± 1.75	Group	4.08	0.043 *	0.013
				Group × Time	0.49	0.485	0.002

*Note.* High_PA = High Physical Activity group; Low_PA = Low Physical Activity group; RT = reaction time; Acc = accuracy. Bold values indicate statistically significant effects (*p* < 0.05). * *p* < 0.05, ** *p* < 0.01, *** *p* < 0.001.

**Table 4:** Multiple comparisons (intra- and inter-group) for executive function outcomes by physical activity group.

Variable	Type	Comparison	*p*	*d*	CI (95%)
Stroop RT (ms)	Inter-group (M1)	High_PA vs. Low_PA	0.024 *	−0.36	[−0.67, −0.05]
	Inter-group (M2)	High_PA vs. Low_PA	0.138	−0.24	[−0.56, 0.08]
	Intra-group	M1 vs. M2 (High_PA)	0.152	−0.19	[−0.51, 0.13]
	Intra-group	M1 vs. M2 (Low_PA)	<0.001 ***	−0.51	[−0.85, −0.17]
Stroop Acc (%)	Inter-group (M1)	High_PA vs. Low_PA	0.068	−0.29	[−0.60, 0.02]
	Inter-group (M2)	High_PA vs. Low_PA	0.377	−0.14	[−0.46, 0.17]
	Intra-group	M1 vs. M2 (High_PA)	0.698	−0.04	[−0.36, 0.28]
	Intra-group	M1 vs. M2 (Low_PA)	0.123	−0.26	[−0.59, 0.07]
PVT-B RT (ms)	Inter-group (M1)	High_PA vs. Low_PA	0.160	−0.23	[−0.54, 0.09]
	Inter-group (M2)	High_PA vs. Low_PA	0.075	−0.29	[−0.61, 0.03]
	Intra-group	M1 vs. M2 (High_PA)	0.167	−0.16	[−0.48, 0.16]
	Intra-group	M1 vs. M2 (Low_PA)	0.453	−0.08	[−0.42, 0.25]
PVT-B Lapses (n)	Inter-group (M1)	High_PA vs. Low_PA	0.025 *	−0.37	[−0.69, −0.05]
	Inter-group (M2)	High_PA vs. Low_PA	0.071	−0.30	[−0.61, 0.02]
	Intra-group	M1 vs. M2 (High_PA)	0.236	0.15	[−0.18, 0.47]
	Intra-group	M1 vs. M2 (Low_PA)	0.913	−0.01	[−0.35, 0.32]
PASAT RT (ms)	Inter-group (M1)	High_PA vs. Low_PA	0.274	−0.17	[−0.48, 0.14]
	Inter-group (M2)	High_PA vs. Low_PA	0.048 *	−0.33	[−0.65, −0.00]
	Intra-group	M1 vs. M2 (High_PA)	<0.001 ***	−0.96	[−1.31, −0.61]
	Intra-group	M1 vs. M2 (Low_PA)	<0.001 ***	−1.07	[−1.44, −0.69]
PASAT Acc (%)	Inter-group (M1)	High_PA vs. Low_PA	0.092	0.27	[−0.04, 0.58]
	Inter-group (M2)	High_PA vs. Low_PA	0.129	0.25	[−0.07, 0.57]
	Intra-group	M1 vs. M2 (High_PA)	<0.001 ***	0.73	[0.39, 1.07]
	Intra-group	M1 vs. M2 (Low_PA)	<0.001 ***	0.74	[0.39, 1.09]

*Note. d* = Cohen’s d; CI = confidence interval. Negative d indicates lower values in the first group/time point. Bold values indicate statistically significant effects (*p* < 0.05). * *p* < 0.05, *** *p* < 0.001.

## Data Availability

The data presented in this study are available on request from the corresponding author. The data are not publicly available due to privacy and ethical restrictions involving minors.

## Abbreviations

The following abbreviations are used in this manuscript:

EF	Executive Function
PA	Physical Activity
BDNF	Brain-Derived Neurotrophic Factor
LMM	Linear Mixed Models
PVT-B	Psychomotor Vigilance Task-Brief
PASAT	Paced Auditory Serial Addition Test
RT	Reaction Time
PPG	Photoplethysmography
ICC	Intraclass Correlation Coefficient
SE	Standard Error
